# The interpretation of systematic reviews with meta-analyses: an objective or subjective process?

**DOI:** 10.1186/1472-6947-8-19

**Published:** 2008-05-21

**Authors:** Ian Shrier, Jean-François Boivin, Robert W Platt, Russell J Steele, James M Brophy, Franco Carnevale, Mark J Eisenberg, Andrea Furlan, Ritsuko Kakuma, Mary Ellen Macdonald, Louise Pilote, Michel Rossignol

**Affiliations:** 1 Centre for Clinical Epidemiology and Community Studies, Lady Davis Institute for Medical Research, SMBD-Jewish General Hospital, McGill University, Montreal, Canada; 2 Department of Epidemiology and Biostatistics, McGill University, Montreal, Canada; 3 Department of Mathematics and Statistics, McGill University, Montreal, Canada; 4 McGill University Health Centre, McGill University, Montreal, Canada; 5 School of Nursing, McGill University, Montreal, Canada; 6 Institute for Work and Health, Toronto, Canada; 7 Department of Oncology, McGill University, Montreal, Canada

## Abstract

**Background:**

Discrepancies between the conclusions of different meta-analyses (quantitative syntheses of systematic reviews) are often ascribed to methodological differences. The objective of this study was to determine the discordance in interpretations when meta-analysts are presented with identical data.

**Methods:**

We searched the literature for all randomized clinical trials (RCT) and review articles on the efficacy of intravenous magnesium in the early post-myocardial infarction period. We organized the articles chronologically and grouped them in packages. The first package included the first RCT, and a summary of the review articles published prior to first RCT. The second package contained the second and third RCT, a meta-analysis based on the data, and a summary of all review articles published prior to the third RCT. Similar packages were created for the 5^th ^RCT, 10^th ^RCT, 20^th ^RCT and 23^rd ^RCT (all articles). We presented the packages one at a time to eight different reviewers and asked them to answer three clinical questions after each package based solely on the information provided. The clinical questions included whether 1) they believed magnesium is now proven beneficial, 2) they believed magnesium will eventually be proven to be beneficial, and 3) they would recommend its use at this time.

**Results:**

There was considerable disagreement among the reviewers for each package, and for each question. The discrepancies increased when the heterogeneity of the data increased. In addition, some reviewers became more sceptical of the effectiveness of magnesium over time, and some reviewers became less sceptical.

**Conclusion:**

The interpretation of the results of systematic reviews with meta-analyses includes a subjective component that can lead to discordant conclusions that are independent of the methodology used to obtain or analyse the data.

## Background

Health care professionals are strongly encouraged to practice evidence-based medicine (EBM) when prescribing treatment for patients [1]. Practicing EBM requires that health care evidence be succinctly summarised for practitioners. A recommended method is the meta-analysis, i.e. the quantitative synthesis and estimation of a summary statistic for the measure of effect [2] based on a systematic review of the literature (also known as a quantitative systematic review). A systematic review with a meta-analysis is often considered the most objective of all types of reviews for the following reasons: a) the search for articles is systematic and extensive, b) inclusion and exclusion criteria are explicitly stated, c) a second reviewer often validates data abstraction, d) data are summarised according to defined methods, and e) an overall summary statistic for the estimate of effect is generated according to accepted statistical methods [3].

Are the processes and interpretation of a systematic review with a meta-analysis objective? Objectivity implies that a person's own beliefs, preferences or attributes should not affect the interpretation of the data. However, meta-analyses performed on the same question by different authors may lead to different conclusions [4]. The discrepancies between meta-analyses are often attributed to the subjective decisions made regarding the procedural issues such as search strategies, inclusion/exclusion criteria, and validity of data abstraction. Because these decisions are subjective and dependent on context, proposed methods of a meta-analysis should be considered standardizations (that increase the transparency and reproducibility of the process) rather than providing an increased level of objectivity. Given the increasing publication of papers describing how to conduct an appropriate meta-analysis [5-8], one might expect that the number of discrepancies between reviews is less now than before. Although this remains to be determined, it is clear that discrepancies persist [9].

Although recommendations on how one should explore the procedural discrepancies between meta-analyses exist [4], little attention has been given to the actual interpretation of the numerical results themselves. Discordant interpretations of the numerical results can be due to variations in knowledge base between readers/authors, but disagreement is also common among professional experts with extensive knowledge [10-14]. Another possibility is that the discrepancies reflect a difference in personal values, resistance to change, and other personal preferences. For example, different clinicians (and different patients) sometimes choose different treatments even when provided with the same options and the same information. Therefore, the objective of this study was to examine the discordance in interpretations amongst eight experienced reviewers when presented with exactly the same data.

## Methods

We presented the same data to eight researchers who had all published systematic reviews and/sor meta-analyses. In brief, these subjects have different professional backgrounds (cardiologist, sport medicine physician, internist, epidemiologist, public health), affiliations (3 different McGill University hospitals; 2 institutions), and years of experience (recent PhD obtained with meta-analysis experience through the Cochrane Collaboration, epidemiologists with 10–25 years experience).

Each reviewer was shown data from randomised trials examining whether intravenous magnesium in the post-myocardial infarction (acute MI) period prevented mortality and arrhythmias. This question was chosen because there was a known discrepancy between the conclusions of meta-analyses and a mega-trial on the topic[15] and one of our interests was whether this feature would differentially affect how reviewers interpreted the meta-analysis. In order to ensure that all reviewers based their decisions on the same information, they were instructed to ignore any knowledge they might have through their personal experience or other readings and to base their responses only on the information provided to them through the review articles, original research articles and meta-analyses. We conducted a systematic review using exhaustive search strategies [magnesium AND (death or mortality or survival) AND (coronary or heart or myocardial or myocardium or cardiac or infarct) AND (randomized or randomised or controlled or trial or double blind or single blind or random or placebo or crossover or RCT)] and searched Medline, Embase, Cochrane Controlled Trials Register, Cumulative Index to Nursing and Allied Health Literature (CINAHL), Science Citation Index, Cochrane Reviews and Database of Abstracts of Reviews of Effects (DARE). We then hand-searched relevant titles from the bibliographies and conducted a citation search on each of the first five published RCTs. Review articles were retrieved with a shortened search strategy omitting the last terms referring to RCTs.

Data were abstracted from the articles by a trained research assistant using standardized data abstraction forms, and verified by a second trained person. Differences were resolved by consensus. We assessed the quality of original manuscripts using the Jadad scale [16,17] and included the information in the reports to the reviewers (there was no a priori exclusion criteria or subgroup analysis). We had initially also used the Chalmers scale [17,18] but abandoned it when the reliability between data abstractors was very poor. After data abstraction, we conducted separate meta-analyses (comparison treatment was always placebo) based on the first RCT, the first 3 RCTs, 5 RCTs, 10 RCTs, 20 RCTs and 23 RCTs. At each time point, the reviewer was given a meta-analysis for mortality, and a separate meta-analysis for arrhythmias. Each meta-analysis included random and fixed effects analyses, a forest plot [8], cumulative forest plot [8], Galbraith plot [19], L'Abbe plot [20] and publication bias statistics and/or plots [8].

The meta-analyses and associated documents were distributed according to a strict protocol. Each reviewer was first given the meta-analysis based on the first RCT and asked a series of questions (see below). Because a meta-analysis should only be interpreted in the context of the strengths and weaknesses of the individual studies and the clinical knowledge at the time, the first meta-analysis was packaged with 1) the completed standardized data abstraction for the first RCT (which included any side effects, co-inverventions, study population, etc described in the original study), 2) summaries of all clinical review articles (to provide for knowledge abut standard of care at the time) and basic science review articles (to provide for knowledge about hypothesized mechanisms and pathophysiology at the time) published before the first RCT (n = 24), and 3) the original publication describing the methods and results of the first RCT addressing the research question. Similarly, the second meta-analysis based on the first 3 RCTs was packaged with the standardized data abstractions and original articles for the 2^nd ^and 3^rd ^RCT, and summaries of all review articles published prior to the 3^rd ^RCT (n = 6). Similar packages were created based on the first 5 RCTs, 10 RCTs, 20 RCTs and all 23 RCTs published at the time data abstraction occurred.

After reading the contents of each package (we did not record the time required but it is estimated between 1–8 hours/package depending on the reviewer and package), each reviewer responded to three clinical questions (Appendix I). First, did the reviewer now consider magnesium a proven effective treatment for an acute MI (answer choices strongly disagree to strongly agree)? Second, did the reviewer believe that magnesium would eventually be proven an effective treatment for an acute MI (answer choices strongly disagree to strongly agree)? Third, would the reviewer recommend magnesium in the treatment of acute MI (answer choices yes or no only)? These questions were chosen because they represent the questions clinicians face on a daily basis, and the types of questions that meta-analyses are supposed to help provide answers to. We restricted answers to Yes/No for the third question regarding recommendation because a clinician must make the decision to either recommend or not recommend treatment (e.g. unsure would mean that treatment is not recommended). This question was especially important because clinical decisions are based on the evaluation of potential benefits and potential harms. Therefore, it is possible to believe a treatment is proven effective for a particular outcome and still not recommend treatment if the side effect profile is not adequately known. It is also possible to recommend a treatment if there is a high probability that the treatment is beneficial even if it has not yet been proven. Reviewers were also encouraged to note specific indications for each question (e.g. conditions where magnesium is beneficial, harmful or unsure), and were also encouraged to write notes to explain their reasoning. We present a qualitative analysis of the data.

## Results

**Figure 1:**
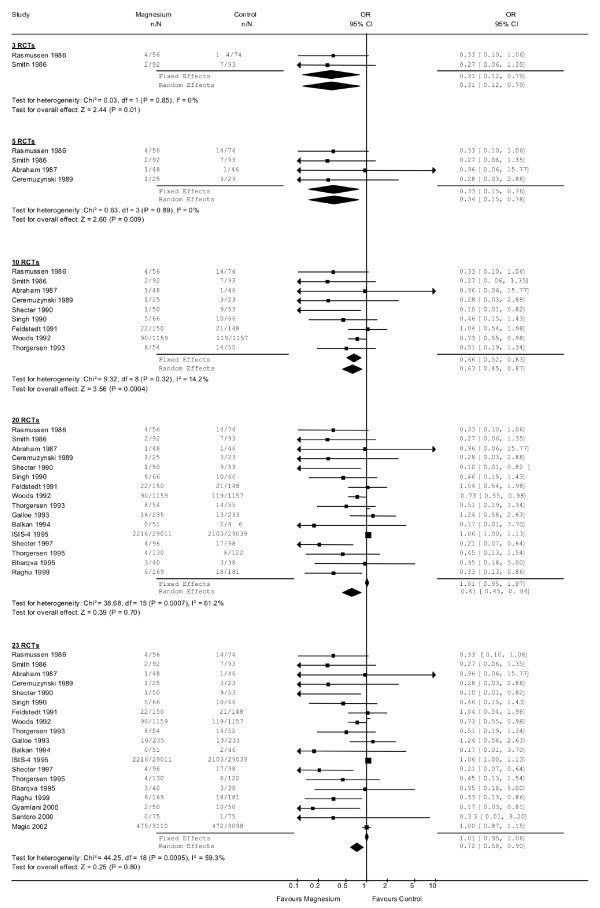
**Forest plots that illustrate the data are shown for each of the meta-analysis packages, along with the fixed and random effects odds ratios at each stage.** The numbers in the graph are slightly different than those found in Table 1 because some data entry errors were discovered at some time points and corrected only after some members reviewed the package. The difference in the numbers between Table 1 and Figure 1 are minor and would not be expected to alter the responses by our reviewers. To remain consistent and avoid confusion, we have provided only the corrected data in Figure 1, and the numbers provided to the reviewers in Table 1. The first error was in Rasmussen 1986 article where the proportions were entered as the raw numbers instead of the frequencies. This was corrected by the next package. A 1988 paper by Rasmussen that was a follow-up analysis based on some criticisms in letters to the editors was included in packages RCT 5 and RCT 10 but then omitted later on. Finally, the numbers for the Thorgersen 1993 paper were correct in the RCT 10 package but were incorrect in the RCT 20 package due to a transcription error when we switched software; this was corrected for the RCT23 package.

We found 23 RCTs that examined the effect of intravenous magnesium on mortality in the early post-myocardial infarction period. The meta-analyses showing the individual studies for each package are shown in Figure 1. The results of the individual meta-analyses are summarised in Table 1, along with the answers from our reviewers. The fixed and random effects odds ratios (OR) were similar for the meta-analyses based on 3 RCTs, 5 RCTs and 10 RCTs, and each result was statistically significant. The meta-analysis based on the first 20 RCTs (5^th ^package) included the large ISIS-4 trial of approximately 50,000 subjects that showed no effect [21]. At this point, the OR based on fixed effects models suggested no effect whereas the OR based on the random effects model suggested a statistically significant benefit (which is expected to occur whenever a very large trial shows no effect and smaller trials show an effect). Similar results were obtained for the last package (23 RCTs).

**Table 1:** Decisions of the 8 reviewers based on the 6 systematic reviews with meta-analyses presented to them.

# RCTS	1	1–3	1–5	1–10	1–20	1–23
N	111	415	597	3685	63047	69505
Fixed OR	N/a	0.40 (0.19–0.83)	0.40 (0.28–0.61)	0.64 (0.52–0.79)	1.02 (0.96–1.08)	1.01 (0.96–1.07)
Rand OR	N/a	0.40 (0.18–0.86)	0.38 (0.21–0.66)	0.66 (0.53–0.81)	0.65 (0.48–0.87)	0.75 (0.61–0.92)
I^2^	N/a	0%	0%	21%	59%	59%
I believe magnesium has now been shown to be beneficial for patients during the post-MI period						
(C)	Disagree	Unsure	Unsure	Disagree	Disagree	Disagree
(C)	Strongly Disagree	Unsure	Unsure	Unsure	Disagree	Strongly Disagree
(P)	Strongly Disagree	Agree	Agree	Agree	Agree	Agree
(P)	Agree	Agree	Strongly Agree	Strongly Agree	Strongly Agree	Agree
(P)	Strongly Disagree	Strongly Disagree	Disagree	Unsure	Agree	Agree
(P)	Unsure	Agree	Agree	Agree	Agree	Agree
(NP)	Strongly Disagree	Disagree	Disagree	Agree	Disagree	Strongly Disagree
(N)	Unsure	Agree	Agree	Agree	Agree	Agree
I believe magnesium will eventually be shown to be beneficial for patients during the post-MI period						
(C)	Agree	Agree	Agree	Agree	Unsure	Disagree
(C)	Unsure	Unsure	Unsure	Unsure	Strongly Disagree	Strongly Disagree
(P)	Unsure	Agree	Agree	Strongly Agree	Agree	Agree
(P)	Agree	Strongly Agree	Strongly Agree	Strongly Agree	Strongly Agree	Agree
(P)	Strongly Disagree	Strongly Disagree	Disagree	Unsure	Agree	Agree
(P)	Agree	Agree	Agree	Agree	Agree	Agree
(NP)	Unsure	Unsure	Unsure	Agree	Unsure	Disagree
(N)	Agree	Agree	Agree	Agree	Agree	Agree
I recommend that magnesium therapy be used in patients during the post-MI period						
(C)	No	No	No	No	No	No
(C)	No	No	No	No	No	No
(P)	No	Yes	Yes	Yes	Yes	Yes
(P)	No	Yes	Yes	Yes	Yes	Yes
(P)	No	No	No	No	No	No
(P)	No	Yes	Yes	Yes	Yes	Yes
(NP)	No	No	No	Yes	No	No
(N)	No	Yes	Yes	Yes	Yes	Yes

C: cardiologist, P: other physician, NP: non-practicing physician, N: non-physician

Each column contains the answers from different meta-analyses based on the number of randomized trials provided (top row). The total number of subjects in each of the meta-analyses is shown in the second row; the overall fixed effects odds ratio (OR) and random effects OR shown to the reviewers are given in rows 3 and 4 (the first trial only examined infarct size and there is no OR for mortality); and the I^2 ^value for heterogeneity is shown in row 5. There were three errors that were discovered after some reviewers had answered questions. The differences in the overall effect estimates were relatively minor and would not be expected to alter the responses by our reviewers. To remain transparent, we provide the numbers provided to the reviewers in this table, and the corrected numbers in Figure 1. The results for each question asked are shown in the subsequent rows. The choices for the first two questions were strongly disagree to strongly agree, and the choices for the third question were yes or no. In addition to the range of interpretations for any one meta-analysis, reviewer 3 moved from unsure to strongly disagree over the 6 meta-analyses for the second question whereas reviewer 7 moved in the opposite direction from strongly disagree to agree.

There was considerable heterogeneity in the reviewers' interpretations for each meta-analysis for all three questions. Even after 10 RCTs with a total of 3685 patients, with similar effect sizes for both random-effects and fixed effects (OR approximately 0.65 (random effects 95%CI: 0.53, 0.81)) and only mild heterogeneity (I^2 ^= 21%), 1 reviewer strongly agreed the treatment was effective, 4 reviewers agreed it was effective, 2 reviewers were unsure and 1 reviewer disagreed the treatment was effective. The discrepancies increased after 20 RCTs, when heterogeneity increased and the OR from the fixed effects and random effects models diverged; 1 reviewer strongly agreed the effect was beneficial, 4 reviewers agreed it was beneficial, and 3 reviewers disagreed it was beneficial. Similar discrepancies were observed when the reviewers were asked if they believed the treatment would eventually be proven beneficial. Finally, when asked if they would recommend the treatment, 4 reviewers fairly consistently said yes (excluding the meta-analysis based on 1 RCT), and 4 reviewers fairly consistently said no.

In addition to the discrepancies related to any one individual meta-analysis, we examined the patterns of responses across meta-analyses per reviewer. From the meta-analysis based on 1 RCT to the meta-analysis based on 10 RCTs, most reviewers' answers to the question whether magnesium has been shown to be beneficial moved from a more negative belief to a more positive belief, with one exception. Between the meta-analysis based on 10 RCTs and the meta-analysis based on 23 RCTs, 3 reviewers became more negative about the treatment, 4 did not change their minds and 1 reviewer became more positive about the treatment.

Although there is not enough power for a statistical analysis, a qualitative examination of the professional background of the reviewers does not suggest any strong tendencies. In general, the cardiologists were negative towards the use of magnesium, as was the non-practicing physician (non-cardiologist). Among the four non-cardiologist physicians, all were generally positive, but one would still not recommend its use.

We also reviewed all the comments made by reviewers but the data did not lend itself to a formal qualitative review. We therefore focused on the comments at 10RCTs (beneficial effect with tight confidence intervals) and 20RCTs (after the large ISIS-4 trial suggested no effect). Of the three reviewers who were the least in favour of magnesium, one remarked that he was almost ready to start recommending magnesium after 10 RCTs but most of the trials were small and therefore he preferred to have one more large study. The other felt that the effect at 10 RCTs was unreasonably large, and probably reflected publication bias. The results of the ISIS-4 trial then reversed any consideration of a real effect for these reviewers. Of those who generally recommended magnesium use, small sample sizes up to the 10RCTs were also a concern and a couple of reviewers limited the recommendation to those individuals not receiving thrombolysis based on the mechanism of action discussed in the review articles. These opinions did not change at 20 RCTs. There were no comments that suggested reviewers incorporated knowledge which came from sources outside the information provided to them.

## Discussion

Although systematic reviews with meta-analyses are considered more objective than other types of reviews, our results suggest that the interpretation of the data remains a highly subjective process even among reviewers with extensive experience conducting meta-analyses. The implications are important. The evidence-based movement has proposed that a systematic review with a meta-analysis of RCTs on a topic provides the strongest evidence of support and that widespread adoption of its results should lead to improved patient care. However, our results suggest that the interpretation of a meta-analysis (and therefore recommendations) are subjective and therefore depend on who conducts or interprets the meta-analysis.

Previous authors examining discrepancies among meta-analyses focused on the subjective decisions regarding procedural issues leading to different data rather than on the interpretation of the data [4]. We presented reviewers with the same meta-analyses and therefore the differences were due to the actual interpretation of the data. The GRADE group also found a lack of consensus among reviewers presented the same data. However, they concluded that this was because some reviewers thought there was sparse data and some did not [22]. Our reviewers disagreed even when there were 10 RCTs with a total of 3685 patients and homogeneity between studies. Further, we minimized the effect of content knowledge by providing a summary of the clinical review articles to all reviewers, and instructing all reviewers to base their decisions only on the information provided in the packages; there was no indication in their written comments suggesting that these instructions were not followed. Even if the reviewers did not follow these instructions, the results would still imply that conclusions are highly dependent on the professional training of the authors (e.g. differences in understanding of physiology, epidemiology). For example, the two cardiologists in our group were generally more sceptical of the effects of treatment. Our study design did not allow for a detailed analysis of the underlying reasons for these results and we plan to explore these more fully in a mixed-methods design in the future. Finally, even though we provided everyone with the quality score of reporting for each study based on the Jadad scale [16], it is possible that the reviewers differentially judged the quality of the studies.

Evidence-based medicine requires that the clinician make decisions based on the numerical results observed. Decision-making and clinical reasoning are complex processes, and different clinicians (and different patients) often choose different treatments even when provided with the same options and the same information. Our results may simply reflect the same process in the context of meta-analysis. There is a large body of literature examining these processes in other areas, and similar processes may be occurring in the meta-analysis context. Some selected examples of different frameworks are illustrated below.

In Gestalt intuition, a subject's decisions are influenced by the identification of hidden relationships within the whole context [23]. With reflective practice, experts adapt their governing analytical premises to the complex problem at hand [11-13,24]. In decision theory, the expert attempts to calculate the probabilities of various outcomes within a specific calculative framework [25,26]. With tacit knowledge, the expert uses implicit decisional processes of which he/she is not consciously aware [27]. The Bayesian approach recognizes that decision-making is a two-step process. The decision-maker first decides what he/she considers is an appropriate estimate for the effect of the treatment, which is dependent on prior beliefs and the likelihood function (fixed or random effects model). In the second step, the decision maker must recognize that there is a risk associated with whatever decision is taken. Therefore, the decision maker weighs 1) the risks of doing harm if they choose to give a treatment they believe is beneficial and the treatment is actually harmful, against 2) the risks of not providing benefit if they choose not give a treatment that they believe is ineffective/harmful and the treatment is actually effective (this is called the loss function in Bayesian analysis).

Our results suggest that a systematic review with a meta-analysis must be viewed with the perspective that it represents one study conducted by specific investigators with a specific methodology. At each step of the methodology (defining the general criteria, search strategy, inclusion/exclusion criteria, data abstraction, and analysis), subjective decisions are required that could affect the validity of the study; the relative importance of each will likely depend on the topic of inquiry and the data acquired. Our study demonstrates that disagreements in the conclusions of systematic reviews with meta-analyses can also be due to subjective interpretations of the results and not only of the methodology. Understood in this context, meta-analyses represent one more source of additional information that allows the scientific community to better understand a clinical question. It must therefore be read with as much caution as any other scientific paper.

Our study has potential limitations. Each of our reviewers had extensive experience conducting systematic reviews with meta-analyses. We believe that reviewers with less experience may interpret data very differently and we will study such reviewers in the future. Other investigators might have abstracted the data differently than us, or used different types of analyses (e.g. risk differences instead of odds ratios). However, this would not affect our results as all reviewers still viewed the exact same data and the actual "true" effect of magnesium on the outcome is not important for the purpose of this study. That said, a second trained individual validated all abstracted data and differences were resolved by consensus. Different reviewers may prefer different models and rely on different types of analyses and plots. Therefore, we provided each reviewer with results based on both fixed and random effects models, publication bias statistics/plots, forest plots, Galbraith plots, L'Abbe plots, and the original article. If a reviewer requested a specific plot or subgroup analysis, this was provided. Our data represents heterogeneous interpretations from one topic only and we cannot generalize to other topics or estimate the frequency with which this might occur. However, we believe this topic was particularly suited to our objective because it allowed us to compare decisions between and within reviewers for meta-analyses with little heterogeneity, and large amounts of heterogeneity. As we previously stated, reviewers were asked to base their decisions only on the information provided in the packages and we cannot be sure if this occurred. Although we asked reviewers to make comments on each package, a formal qualitative analysis was not possible with the data obtained.

Finally, although our data suggest that different reviewers interpret data differently, this study cannot provide insight into the reasons why. The answers to this very important question likely include subtleties and nuances that are difficult to capture using quantitative methods, and we will examine these subjective elements in a future study using a mixed-methods approach.

## Conclusion

The interpretation of systematic reviews with meta-analyses is at least partially subjective. Evidence-based practitioners need to be aware that any conclusions and recommendations based on a systematic review with a meta-analysis should be read with caution even if the methodology is rigorous.

## Competing interests

The authors declare that they have no competing interests.

## Authors' contributions

IS developed the original idea, supervised the study, and wrote the first draft of the paper. J–FB, RWP, RJS, JMB, AF, RK and MR all helped develop the ideas while the grant was being written, and have provided comments on the analysis and the manuscript. FC, MJE, MEM and LP all helped with the analysis stages, and with writing the manuscript. All authors have given approval for the final version of the manuscript.

## Pre-publication history

The pre-publication history for this paper can be accessed here:


